# Introducing a new reporter gene, membrane-anchored *Cypridina* luciferase, for multiplex bioluminescence imaging

**DOI:** 10.1016/j.omto.2021.03.004

**Published:** 2021-03-17

**Authors:** Maxim A. Moroz, Juan Zurita, Anna Moroz, Ekaterina Nikolov, Yury Likar, Konstantin Dobrenkov, Jason Lee, Larissa Shenker, Ronald Blasberg, Inna Serganova, Vladimir Ponomarev

**Affiliations:** 1 Department of Radiology, Memorial Sloan Kettering Cancer Center, New York, NY 10065, USA; 2 Department of Neurology, Memorial Sloan Kettering Cancer Center, New York, NY 10065, USA; 3 Molecular Pharmacology and Chemistry Program, Memorial Sloan Kettering Cancer Center, New York, NY 10065, USA; 4 Skolkovo Institute of Science and Technology, Moscow 143026, Russia

**Keywords:** bioluminescence imaging, BLI, luciferase reporters, reporter gene, multiplex imaging

## Abstract

Bioluminescence reporter gene imaging is a robust, high-throughput imaging modality that is useful for tracking cells and monitoring biological processes, both in cell culture and in small animals. We introduced and characterized a novel bioluminescence reporter—membrane-anchored *Cypridina* luciferase (maCLuc)—paired with a unique vargulin substrate. This luciferase-substrate pair has no cross-reactivity with established d-luciferin- or coelenterazine-based luciferase reporters. We compare maCLuc with several established luciferase-based reporter systems (firefly, click beetle, *Renilla*, and *Gaussia* luciferases), using both *in vitro* and *in vivo* models. We demonstrate the different imaging characteristics of these reporter systems, which allow for multiplexed-luciferase imaging of 3 and 4 separate targets concurrently in the same animal within 24 h. The imaging paradigms described here can be directly applied for simultaneous *in vivo* monitoring of multiple cell populations, the activity of selected signal transduction pathways, or a combination of both constitutive and inducible reporter imaging.

## Graphical abstract

**Figure:**
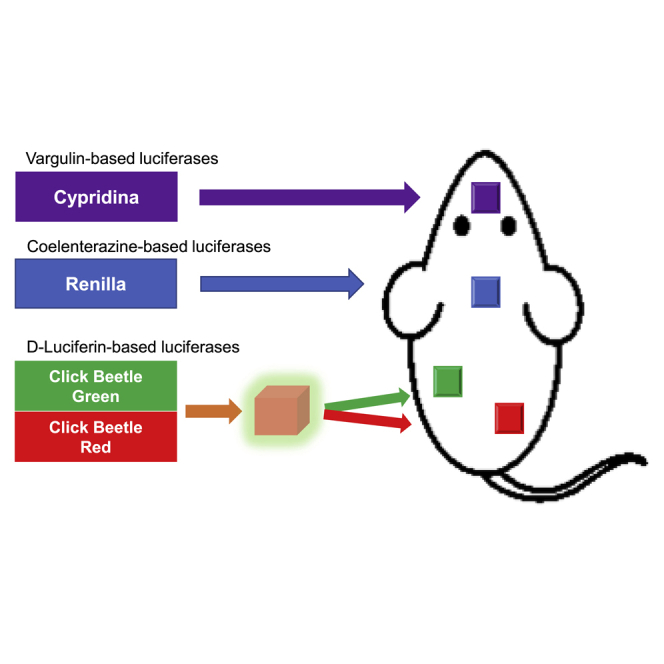

## Teaser

Moroz et al. developed and tested a new reporter system based on a membrane-anchored *Cypridina* luciferase gene. maCLuc in combination with other established BLI reporter systems provides the opportunity for a new, multiplex imaging paradigm. This paradigm will allow scientists to visualize up to 3 and 4 separate targets concurrently in the same animal.

## Introduction

Bioluminescence reporter imaging (BLI) is a frequently used imaging tool in biology and cancer research for monitoring tumor development, formation of metastases, and cell trafficking in animal models with “constitutive” reporters and for monitoring cell function and signal pathway activation with “inducible” reporters. BLI is a highly robust, reliable, and sensitive imaging modality that lends itself to high throughput and is relatively low cost. It is applied throughout the field of biotechnology, from fundamental research to validation of novel clinically applicable therapeutics.

Despite recent advances in novel BLI reporter gene development, there are only two well-established major classes of the luciferases, d-luciferin-based (most commonly firefly and click beetle red and green luciferases) and coelenterazine-based (wild-type and mutant forms of *Renilla* and *Gaussia* luciferases) luciferases that allow simultaneously monitoring up to two cell populations *in vivo*. Recently, a new class of bioluminescence reporters derived from *Vargula hilgendorfii* and *Cypridina noctiluca* marine ostracods^1^^,^^2^ has been described, which utilize a unique substrate (vargulin). Both *Vargula* and *Cypridina* luciferase enzymes are naturally secreted from their respective hosts and catalyze oxidation of vargulin, resulting in blue light emission (465 nm). Importantly, these luciferases do *not* cross-react with d-luciferin or coelenterazine, nor do the conventional luciferases react with vargulin.

In this report, we describe the successful development of a new BLI reporter suitable for whole body imaging—a membrane-anchored *Cypridina* luciferase (maCLuc). This new maCLuc BLI reporter (coupled with the unique vargulin substrate) possesses superior emission characteristics for multiplexing with d-luciferin- and coelenterazine-based luciferases. We propose a simple and highly reproducible paradigm to image three different cell populations or molecular biological processes (both *in vitro* and *in vivo*) using three independent substrates. Additionally, we provide insights for further expansion, namely, to successfully integrate spectral separation of the same-substrate luciferases for simultaneous BLI of four independent cell populations *in vivo*.

## Results

### *In vitro* characterization of the membrane-anchored *Cypridina* luciferase and a comparison with other luciferases

**Figure 1:**
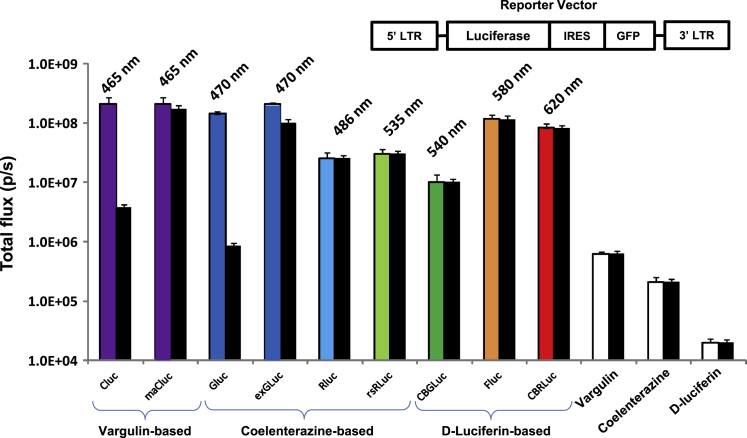
*In vitro* assessment of luciferase reporters *In vitro* BLI of different luciferases comparing signal intensity of total (both cellular and media: color bars) and cellular alone (black bars) different reporter-transduced C6 cell lines. Photon flux of the different reporter cell lines was normalized by GFP tag expression. The structure of the reporter vector is shown. Data shown as averages from 3 separate studies (total of 9 samples) with standard deviations (SDs) < 10%.

Since native *Cypridina* luciferase (CLuc) is secreted (exported from the cell), the photons are emitted predominantly from the cell culture medium. An N-terminally truncated CLuc mutant anchored at its C terminus to the cell surface membrane with a transmembrane domain from human low-affinity nerve growth factor receptor (LNGFR; named membrane-anchored CLuc, maCLuc) was developed and used for further analysis (Figure S1). We compared the bioluminescence intensity of different reporter systems, normalized to the level of green fluorescent protein (GFP) expression, as well as the kinetics of light output using the appropriate substrate (Figure 1). Native (secreted) forms of *Gaussia* luciferase (GLuc) and CLuc demonstrated superior bioluminescent signals, with a high total flux of 2.1 × 10^8^ and 2.2 × 10^8^ photons/s per 10,000 cells, respectively. While ~90% of the native CLuc and GLuc enzymes were secreted into the culture medium, we observed only 5% and 10% secretion from the maCLuc- and externalized *Gaussia* luciferase (extGLuc)-transduced cells, respectively. Of note, both maCLuc and extGLuc demonstrated high total signal outputs (2.1 × 10^8^ and 1.9 × 10^8^ photons/s, respectively). Widely used d-luciferin-based luciferases—such as firefly (FLuc), click beetle red (CBRLuc), and green (CBGLuc)—also showed robust signals in our comparative assay (1.2 ± 0.2 × 10^8^, 1.03 ± 0.11 × 10^8^, and 1.1 ± 0.12 × 10^7^ photons/s, respectively) and are not secreted. The coelenterazine-based luciferases—*Renilla* (RLuc) and red-shifted *Renilla* (rsRLuc)—had slightly lower signal intensities (2.1 ± 0.4 × 10^7^ and 2.3 ± 0.3 × 10^7^ photons/s) but no evidence of secretion into the media. The *in vitro* kinetics of light output were consistent with the published literature for the respective substrates.^3^

### *In vivo* bioluminescence imaging: comparison of different luciferases

**Figure 2:**
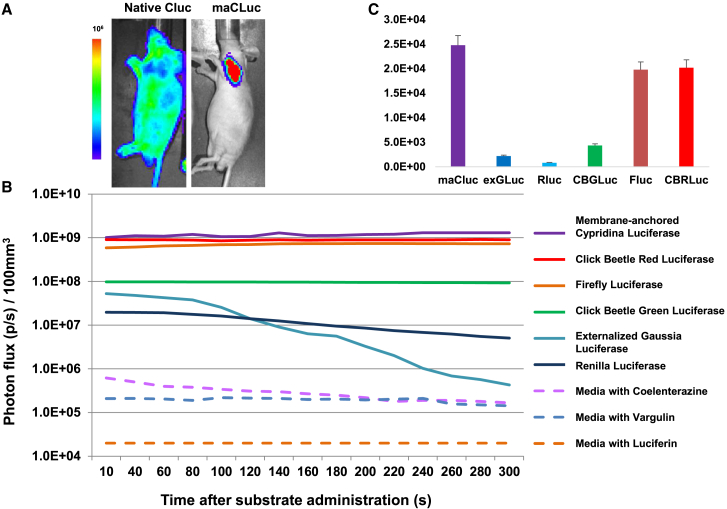
*In vivo* imaging of BLI reporters (A) BLI imaging of C6-derived xenografts transduced with native and externalized forms of *Cypridina* luciferase. Imaging was performed 30 s after administration of CLuc-specific substrate vargulin. (B) Dynamic *in vivo* BLI of subcutaneous xenografts (solid lines) derived from different reporter-transduced C6 cell lines (solid lines) and substrate-specific background (dashed lines). Photon flux from the different reporter-transduced tumors was normalized for tumor volume (per 100 mm^3^). (C) Signal-to-noise (animals injected with substrate only) ratios for *in vivo* BLI imaging of C6-derived xenografts transduced with different luciferases. n = 9 for each luciferase.

Luciferase-transduced C6 xenografts were established by subcutaneous injection of 1 × 10^6^ cells in the shoulders and thighs of the animals. The signal intensity assessment, spectral analysis, and comparison were performed with the available emission filters (500–800 nm) on an IVIS Spectrum Imaging System (Caliper). Imaging was performed 7 days after implantation of the xenografts. The i*n vivo* assessment showed a strong focal BLI signal coming from maCLuc-expressing tumors, while a diffuse whole-animal bioluminescence was observed in mice bearing native CLuc xenografts, due to secretion of CLuc from C6 cells (Figure 2A). Tumors derived from C6/maCLuc^+^ cells showed the highest signal among the luciferases tested (1.2 × 10^9^ ± 6% photons/s, normalized to tumor volume, Figure 2B). Dynamic imaging demonstrated the stability of the signal for d-luciferin- and vargulin-based luciferases (up to 15 min post injection). Xenografts with the externalized form of GLuc (extGLuc) showed a high BLI signal at early time points after administration of substrate (30–60 s), followed by a rapid decrease (reaching background levels in 4 min). RLuc-bearing xenografts showed moderate signal levels. A high substrate-normalized sensitivity (3–5 log photons/s) was observed for d-luciferin-based luciferases, compared to ~3 log photons/s for maCLuc and a 1.5–2 log photons/s sensitivity for coelenterazine-based reporters (Figure 2C; Table 1).

**Table 1:** Substrate-normalized luciferase signal intensity comparison

Luciferase	Sensitivity ratio
CBGLuc	4.35 × 10^3^
CBRLuc	2.02 × 10^4^
maCLuc	2.48 × 10^4^
RLuc	8.12 × 10^2^
FLuc	1.98 × 10^4^
extGLuc	2.19 × 10^3^

### Triple-multiplex bioluminescence *in vivo* imaging

**Figure 3:**
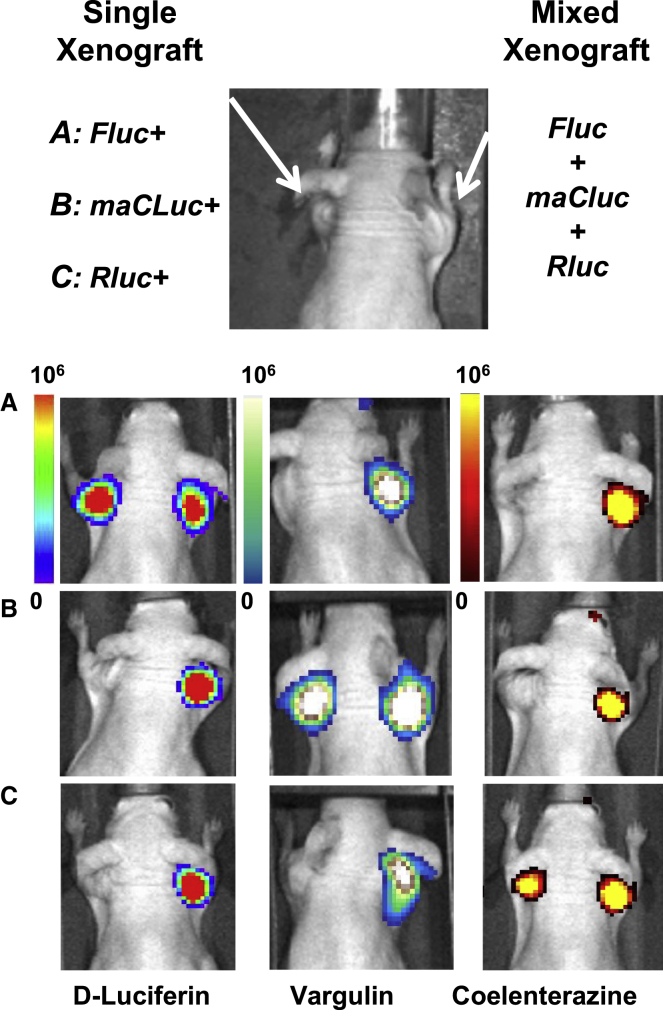
Triple luciferase imaging *in vivo* Triple luciferase imaging in the same animal using 3 independent reporter-substrate combinations. All the animals bear a xenograft derived from 1:1:1 mixed C6 cells with CBRLuc, maCLuc, and RLuc reporter genes on the right shoulder. (A–C) On the left shoulder, animals had xenografts derived from a single C6 line transduced with the FLuc (A), maCLuc (B), or RLuc (C) reporter. Three different luciferase substrates (d-luciferin, vargulin, and coelenterazine; vertical columns) were injected i.v. into each animal (A–C), and imaging was performed sequentially with a 4- to 12-h interval between sessions (n = 5 for each group).

To perform concurrent/sequential BLI of three different cell populations (or biological processes) *in vivo*, we included the maCLuc reporter, since it can be visualized with a unique substrate, vargulin. This luciferase-substrate pair has no cross-reactivity with established d-luciferin- or coelenterazine-based luciferase reporters and allows for triple-luciferase imaging in the same animal. To test the efficacy of triple-reporter BLI, three groups of animals bearing xenografts derived from a single C6 line transduced with CBRLuc, maCLuc, or RLuc reporter and 1:1:1 mixed CBRLuc/maCLuc/RLuc C6 cells were compared (Figure 3). These three separate reporter systems were successfully imaged within a 24-h period without cross-contamination using the three luciferase-substrate combinations. A 4- to 12-h period between imaging sessions was required to allow for substrate decay (4 h for coelenterazine, 6–8 h for vargulin, 8–12 h for d-luciferin).

**Figure 4:**
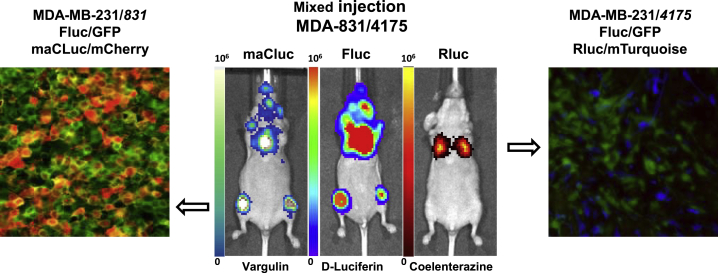
Imaging of secondary tumor development Three weeks after administration of bone-avid MDA-MD-231/*831* (FLuc/GFP-maCLuc/mCherry) and lung-avid MDA-MB-231/*4175* (FLuc/GFP-RLuc/mTurquoise) human breast cancer cell lines, BLI images (middle panel) were obtained using three independent substrates: vargulin, d-luciferin, and coelenterazine, which are specific for maCLuc, FLuc, and RLuc, respectively. Immunofluorescence analysis of an MDA-231-831 bone metastasis (GFP/mCherry, left panel) and an MDA-231-4175 lung metastasis (GFP/mTurquoise, right panel). Each experiment was repeated three times (n = 5 for each group).

To further investigate the advantage of multi-luciferase imaging in the same animal, we have used breast cancer cell lines (MDA-231-831 and MDA-231-4175) expressing the FLuc/GFP fusion reporter (PMID: 28322342). These tumor models tend to form metastases selectively in bones (MDA-231-831) or lungs (MDA-231-4175) (Figure 4). The cell lines were transduced with the newly developed maCLuc/mCherry and RLuc/mTurquoise vectors, respectively (Figure S2). A mixture of MDA-231-831-FLuc/GFP^+^maCLuc/mCherry^+^ and MDA-231-4175-FLuc/GFP^+^Rluc/ mTurquoise^+^ cell populations were administered by intracardiac or intravenous (i.v.) injection, to mimic hematogenic metastatic dissemination. Using the FLuc/d-luciferin BLI readout, we observed the formation of tumors in thighs, skull, and chest of the animals (Figure 4). Then, using maCLuc/vargulin and RLuc/coelenterazine readouts, we were able to differentiate between MDA-231-831-specific (sternum, femur, and skull) and MDA-231-4175-specific (lungs) metastatic sites. *Ex vivo* fluorescence analysis of BLI-positive tissues corroborated the *in vivo* imaging data and allowed for clear visualization of MDA-231-831 GFP/mCherry double-positive metastases in bones and MDA-231-4175 tumors expressing GFP and mTurquoise in lung tissues (Figure 4).

### Quadruple-multiplex bioluminescence *in vivo* imaging

**Figure 5:**
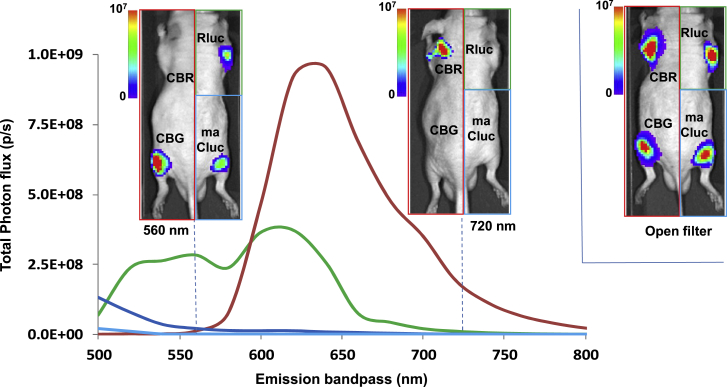
*In vivo* BLI of subcutaneous luciferase-expressing xenografts using a spectral band-pass protocol Three separate/sequential reporter/substrate-specific BLI sessions were performed in the same animal. Each animal bore 4 different reporter-transduced s.c. C6 tumors: (1) RLuc^+^, right shoulder; (2) maCLuc^+^, right thigh; (3) CBRLuc^+^, left shoulder; and (4) CBGLuc^+^, left thigh. Three different luciferase substrates were administered i.v. at different times over a 24-h period: coelenterazine (for RLuc) at t = 0 h, vargulin (for maCLuc) at t = 6 h; and d-luciferin (for CBRLuc and CBGLuc) at t = 18 h. BLI was performed with 10 narrow band-pass filters (560 nm, left panel; 720 nm, center panel) or with an “open filter” (500–800 nm, right panel). Three separate BLI studies in the same mouse are shown in the composite images: CBRLuc^+^ and CBGLuc^+^ tumors with d-luciferin (red border, left image); RLuc^+^ tumor with coelenterazine (green border, right upper image); maCLuc with vargulin (blue border, right lower image). The spectra of CBRLuc (red line, peak 640 nm) and CBGLuc (green line, peaks at 550 nm and 610 nm) are shown; the blue-emitting RLuc (486 nm peak) and maCLuc (465 nm peak) were outside of the spectral analysis range. The spectral band-pass imaging shows spectral separation between CBGLuc- and CBRLuc-expressing xenographs at 560 nm (left panel) and 720 nm (center panel), respectively (n = 5 for each group of mice).

In the next series of experiments, the capabilities of sequential multiplex BLI imaging were explored further. Four independent cell populations (transduced with four different constitutively expressed luciferases: maCLuc^+^, CBRLuc^+^, CBGLuc^+^, and RLuc^+^) were implanted in the same animal. An IVIS Spectrum with a range of light wavelength-blocked filters (500–800 in 20-nm increments) was used to obtain a spectral separation of the BLI signals emanating from the four different xenografts. Image separation was achieved by using three different substrates and applying spectral unmixing to the images (Figure 5). Spectral imaging demonstrated a bi-phasic maximum intensity peak (MIP) for CBGLuc at 550 nm and 610 nm (green line) and a mono-phasic peak at 640 nm for CBRLuc (red line). Spectral unmixing provided spectral separation of the signals derived from the two d-luciferin-based reporters: separation of the CBGLuc reporter (imaged at 560 nm and below) from the CBRLuc reporter (imaged at 720 nm and above). MIPs for blue-emitting RLuc (486 nm) and maCLuc (465 nm) were outside of the spectral analysis range because of the lack of an available lower-wavelength filter (blocking wavelengths below 500 nm). It was clearly demonstrated that coelenterazine and vargulin have no cross-reactivity with each other or d-luciferin, which allows for quadruple-luciferase imaging in the same animal.

## Discussion

This study had two aims: (1) to develop and evaluate a novel bioluminescence reporter system for *in vivo* imaging, maCLuc, compared to established BLI reporter systems, and (2) to validate the feasibility of multiplex (triple and quadruple) noninvasive bioluminescence reporter gene imaging using the different substrate specificity and spectral characteristics of the BLI reporters.

Until recently there were only two classes of BLI reporters: d-luciferin- and coelenterazine-based luciferases (e.g., FLuc and RLuc) that allowed for simultaneous monitoring of two independent cell populations or molecular processes *in vivo*.^4^^,^^5^ Later, dual-spectral luciferases based on bioluminescent enzymes with non-overlapping luminescence spectra were described that can be imaged simultaneously by filter-based detection within the same organism using a single reagent (e.g., d-luciferin for CBRLuc and CBGLuc).^6^

We have explored the feasibility of triple and quadruple *in vivo* BLI using a new class of BLI reporters—vargulin-based luciferases. CLuc is a secreted bioluminescent protein cloned from the ostracod *Cypridina noctiluca*, which catalyzes the oxidation of its unique substrate vargulin, resulting in photon emissions with a wavelength peak at 465 nm.^7^ When expressed in transduced cells in its native form, CLuc is secreted;^8^ therefore, whole-body BLI is suboptimal since the secreted luciferase results in a whole-body bioluminescence (Figure 2A). Based on prior work and publications,^9^ we engineered a maCLuc that has a predominant localization at the outer membrane of transduced cells with minimal secretion (Figures 1 and 2; Figure S1). The maCLuc reporter demonstrated a very high bioluminescent signal recorded both *in vitro* (Figure 1) and *in vivo* (Figure 2) and has high substrate-normalized sensitivity compared to other luciferases when a wide spectral window is used in the acquisition of emitted photons (Figure 2C; Table 1). The distinctive features of the maCLuc reporter allow for successful multiplexing with d-luciferin- and coelenterazine-based luciferases for imaging different cell populations within the same animal by using three independent substrates.

Unlike luciferases exhibiting flash kinetics (high initial light intensity emission followed by rapid decay, e.g., RLuc, GLuc),^10^ maCLuc bioluminescence is a more extended, glow type of light emission. This makes maCLuc a desirable reporter for imaging and various bioluminescence assays, specifically allowing for the time-resolved, multiplexed detection of multiple targets. Indeed, dynamic BLI of different luciferase-expressing xenographs showed a very stable (up to 15 min) signal from maCLuc and d-luciferin-based enzymes, while the signal from coelenterazine-based flash kinetic reporters started to “fade” within the initial 1–3 min after coelenterazine administration (Figure 2B).

The ability of multiplexing different BLI reporters within the same animal was successfully demonstrated with a subcutaneous (s.c.) xenograft model, as well as a systemically administered metastatic tumor model. In both experimental designs, xenographs expressing RLuc, maCLuc, and CBRLuc/CBGLuc/FLuc reporters were successfully visualized with administration of the appropriate substrates (Figures 3 and 4). An optimized order of substrate administration, with an 8- to 12-h gap between imaging sessions, resulted in a BLI protocol that was successfully completed within a 24-h period, without BLI signal cross-contamination or overlap. In addition, spectral imaging with specific light wavelength-blocked filters was successfully applied to obtain a spectral separation of the CBGLuc and CBRLuc signals. The application of spectral imaging in these studies allows for whole-body BLI of four different xenografts (Figure 5) or could be applied to study four different reporter-based biological processes concurrently in the same animal. However, the multiplexing of four BLI reporter systems requires specific wavelength-blocked filters. For the multiplex imaging paradigm described here, filters ranging from 500 to 800 nm with 20-nm intervals would be optimal.

Many pre-clinical models involve administration and subsequent monitoring of cells of different origins, biological phenotype, organ-specific trafficking, and persistence (e.g., isogenic tumors, immune cell sub-populations, engineered therapeutic cells).^11^, ^12^, ^13^, ^14^, ^15^ Similarly, multiple molecular biological processes^16^ that are independent or inter-related would benefit from continuous reporter-based monitoring over time within the same experimental animal. Since BLI is a non-invasive whole-body pre-clinical imaging technique, it can provide valuable information by multiplexing bioluminescence reporters introduced into specific cells or tissues of interest. The imaging paradigms described here can be directly applied to monitor treatment response of multiple tumor cell populations within the same animal;^17^ study the dynamics of immune cells and immune cell sub-populations targeting multiple tumors;^13^ or monitor the activity of selected signal transduction pathways during the course of disease progression and following specific therapies.^18^

The use of a particular BLI reporter should be carefully considered based on the biology of the cells and the experimental design. For example, “weaker” coelenterazine-based reporters can be used for imaging of fast-growing tumors, while imaging of sparsely distributed stem cells or immune cells would benefit from a more potent vargulin- or d-luciferin-based reporter to increase imaging sensitivity. Another important consideration is the depth at which the targeted/transduced cell population is expected to be visualized: the signal from a “blue” luciferase will be significantly attenuated if produced within deep tissues (e.g., liver, pancreas), while the signal from red-shifted enzymes will be more easily detected.^6^ It is worth noting that two or more BLI reporters with similar spectral characteristics (e.g., extGLuc and maCLuc, with different non-cross-reacting substrates, coelenterazine and vargulin) can be particularly valuable in certain situations. For example, when comparing photons emitted from small cell populations (e.g., metastases, immune cell subpopulations) simultaneously at the same anatomical site, differences in the recorded BLI signal would be less influenced by the depth of the light source when the reporter pair wavelengths are similar.

### Conclusions

We developed and tested a new BLI reporter, based on a membrane-anchored *Cypridina* luciferase gene, that showed limited secretion and high light output. We have used the novel maCLuc reporter in combination with established BLI reporter systems to validate pre-clinical imaging paradigms, allowing for multiplex BLI of 3 and 4 separate targets concurrently in the same animal. Our experiments allow adoption of this new powerful reporter system for further *in vivo* imaging applications, both as an individual BLI reporter as well as in combination with other luciferases.

## Materials and methods

### Vector development

All DNA manipulations were performed using restriction enzymes, T4 DNA ligase, CIP, and buffers according to standard procedures and manufacturer’s instructions (New England Biolabs, Ipswich, MA, USA). In order to minimize CLuc secretion from the transduced cells, an N-terminally truncated variant of CLuc was fused at its C terminus to a transmembrane domain from human LNGFR, resulting in a cell surface membrane-anchored CLuc mutant, maCLuc. A retroviral vector encoding constitutively expressed firefly luciferase and GFP separated by an internal ribosome entry site, SFG-FLuc-IRES-GFP, was developed using SFG-hsv1TK-IRES-GFP^19^ plasmid as a backbone. Eight additional -IRES-GFP retroviral vectors were developed by replacing the FLuc gene sequence with other gene sequences coding for CLuc, maCLuc, GLuc, extGLuc, RLuc8, rsRLuc, CBGLuc, and CBRLuc bioluminescent enzymes (Figure 1). For the experiments with breast cancer cells, additional vectors were developed: Rluc-IRES-mTurquoise was developed by replacing GFP in RLuc-IRES-GFP vector with mTurquoise fluorescent protein, and maCLuc-IRES-mCherry was developed by replacing GFP in maCLuc-IRES-GFP vector with mCherry fluorescent protein.

### Cell transduction and fluorescence-activated cell sorting (FACS)

C6 (rat glioma) and MDA-MB-231-4175 and MDA-MB-231-831 (human breast cancer) cell lines were maintained in DMEM media supplemented with 4.5 mM glucose and 10% serum. All cell lines were stably transduced with reporter-encoded retroviral vectors (see above), by incubating 50% confluent cell cultures with virus-containing medium for 12 h in presence of polybrene (8 mg/mL; Sigma, St. Louis, MO, USA). GFP expression in transduced tumor cells was visualized by fluorescence microscopy using a Nikon Eclipse T-100 (Morrell, Melville, NY USA). C6 cells were transduced with all bioluminescence reporter-IRES-GFP constructs for direct comparison. Both MDA-MB-231 cell sublines with different metastatic potentials were transduced with the FLuc-IRES-GFP vector. The MDA-231-MB-4175-line form was than co-transduced with RLuc-IRES-mTurquoise and the MDA-MB-231-831 line with maCLuc-IRES-mCherry. After transduction, the cells were assessed for fluorescent protein expression as previously described.^19^

### *In vitro* assays and BLI

Stably transduced, viable, and sorted cells were seeded in 96-well plates (10,000 cells/well). After incubation of cells overnight, bioluminescence assays were performed in 96-well plates in a 100 μL volume/well with 5 μL of 30 mg/mL (4.7 mmol) d-luciferin stock solution (Fisher Scientific, USA) per well, 1 μL of 2 mg/mL (46.5 μmol) coelenterazine stock solution (NanoLight Technology, USA), or 1 μL of 2 mg/mL (49.3 μmol) vargulin stock solution (NanoLight Technology, USA). All of the substrates were prepared according to vendor specifications. For each cell line, bioluminescence imaging was performed under the following conditions: (1) cells + medium (standard growing conditions), (2) cells + fresh medium (growing medium was aspirated and replaced with fresh medium before imaging), and (3) removed medium (aspirated medium from set 1 was also imaged). Bioluminescence was measured with an IVIS Spectrum Imaging System (Caliper). The acquisition time was 1–180 s, depending on the extent of signal saturation in the different samples. All measurements are reported as total flux normalized by time (photons/s).

### Xenograft models

All animal studies were performed under an animal protocol approved by the Memorial Sloan Kettering Cancer Center (MSKCC) Institutional Animal Care and Use Committee.

C6 xenografts were established by subcutaneous injection of 4 × 10^6^ cells in the shoulders of the athymic rnu/rnu mice (Taconic, NY, USA), 10 animals per group, and used for direct comparison and dynamic imaging. Group 1 contained a FLuc-IRES-GFP xenograft in the left shoulder and a RLuc-IRES-GFP xenograft in the right shoulder. Group 2 contained a CBRLuc-IRES-GFP xenograft in the left shoulder and an extGLuc-IRES-GFP xenograft in the right shoulder. Group 3 had a wild-type C6 xenograft in the left shoulder and a native (wild-type)∗GLuc-IRES-GFP xenograft in the right shoulder. Group 4 animals had a CBG-IRES-GFP xenograft in the left shoulder and a maCLuc xenograft in the right shoulder. Group 5 had a “single” native CLuc xenograft in the left shoulder. Xenografts were allowed to grow for 10 days, and the size of the tumors was assessed every 2 days with caliper measurements. A separate group of animals (n = 5/group) were prepared to demonstrate the triple and quadro imaging approach with cells injected in both shoulders (*Renilla* and CBRLuc) and both thighs (maCLuc and CBGLuc). MDA-231-bearing xenografts were developed based on the selective affinity of the MDA-231-4175 clone to metastasize to the lungs and of the MDA-231-831 clone to metastasize to bone tissue.^20^ MDA-231-4175 cells were administered as a bolus i.v. injection (2 million cells in 0.2 mL of saline). Two weeks later, MDA-231-831 cells were administered via intracardiac injection, 5 × 10^4^ cells in 0.05 mL of saline.

### *In vivo* bioluminescence imaging

Bioluminescence imaging was performed 7 days after implantation of the xenografts with an IVIS Spectrum Imaging System (Caliper), 10–15 s after retro-orbital injection of coelenterazine, vargulin (both 10 μg per animal; NanoLight Technology), or d-luciferin (3 mg per animal; Fisher Scientific). Mice were imaged in pairs over a time course of 5 min, using 5-s acquisitions. A 25-cm field of view, with medium binning and a f-stop of 1, was used during the image acquisitions. Region of interest analysis was performed with Living Image software (Xenogen), and signal intensity was normalized to tumor volume − photon flux (p/s)/tumor volume (100 mm^3^). Tumor volumes were obtained by caliper measurements at the time of imaging –V = (W(2) × L)/2.^21^ Substrate-normalized luciferase signal intensity was calculated as a ratio in log photons/s between the highest BLI signals recorded from a transduced xenograft and the tissue background. To assess the spectral characteristics of the photons emitted, a sequence of 20-nm band-pass images (500–800 nm in visible spectra) was obtained with the IVIS Spectrum filters.

In the animals bearing xenografts with different luciferases, the substrate administration was performed in the following order: coelenterazine followed by vargulin and d-luciferin 8 and 20 h, respectively, after coelenterazine. Prior to imaging with the second and third substrates, a control image acquisition was performed to ensure the absence of the residual signal from the prior substrate.

### Tissue and cell analysis

Histopathologic examination of the specimens and cell pellets was performed as described previously.^3^ For immunofluorescence analysis, freshly isolated tumor and liver tissue were fixed in 4% paraformaldehyde or snap-frozen in OCT compound (Sakura Finetek); 7-μm cryostat tissue sections were then cut for immunofluorescence staining. For the visualization of nuclei, 4′,6-diamidino-2-phenylindole was used. Immunofluorescence was assessed with a fluorescence microscope (MIRAX, Carl Zeiss Microimaging).

### Statistical analysis

Statistical significance of differences between mean values was estimated with Excel (Microsoft) using the independent t test for unequal variances. p values <0.05 were considered to be statistically significant.

## Supplemental information

Document 1. Figures S1 and S2

Document 2. Article plus supplemental information

## References

[1] Nakajima Y., Kobayashi K., Yamagishi K., Enomoto T., Ohmiya Y. (2004). cDNA cloning and characterization of a secreted luciferase from the luminous Japanese ostracod, Cypridina noctiluca. Biosci. Biotechnol. Biochem..

[2] Maguire C.A., Bovenberg M.S., Crommentuijn M.H., Niers J.M., Kerami M., Teng J., Sena-Esteves M., Badr C.E., Tannous B.A. (2013). Triple bioluminescence imaging for in vivo monitoring of cellular processes. Mol. Ther. Nucleic Acids.

[3] Loening A.M., Wu A.M., Gambhir S.S. (2007). Red-shifted Renilla reniformis luciferase variants for imaging in living subjects. Nat. Methods.

[4] Bhaumik S., Gambhir S.S. (2002). Optical imaging of Renilla luciferase reporter gene expression in living mice. Proc. Natl. Acad. Sci. USA.

[5] Fraga H. (2008). Firefly luminescence: a historical perspective and recent developments. Photochem. Photobiol. Sci..

[6] Daniel C., Poiret S., Dennin V., Boutillier D., Lacorre D.A., Foligné B., Pot B. (2015). Dual-Color Bioluminescence Imaging for Simultaneous Monitoring of the Intestinal Persistence of Lactobacillus plantarum and Lactococcus lactis in Living Mice. Appl. Environ. Microbiol..

[7] Thorne N., Inglese J., Auld D.S. (2010). Illuminating insights into firefly luciferase and other bioluminescent reporters used in chemical biology. Chem. Biol..

[8] Inouye S., Ohmiya Y., Toya Y., Tsuji F.I. (1992). Imaging of luciferase secretion from transformed Chinese hamster ovary cells. Proc. Natl. Acad. Sci. USA.

[9] Santos E.B., Yeh R., Lee J., Nikhamin Y., Punzalan B., Punzalan B., La Perle K., Larson S.M., Sadelain M., Brentjens R.J. (2009). Sensitive in vivo imaging of T cells using a membrane-bound Gaussia princeps luciferase. Nat. Med..

[10] Kim J.E., Kalimuthu S., Ahn B.C. (2015). In vivo cell tracking with bioluminescence imaging. Nucl. Med. Mol. Imaging.

[11] Huang N.F., Okogbaa J., Babakhanyan A., Cooke J.P. (2012). Bioluminescence imaging of stem cell-based therapeutics for vascular regeneration. Theranostics.

[12] Hong H., Yang Y., Zhang Y., Cai W. (2010). Non-invasive cell tracking in cancer and cancer therapy. Curr. Top. Med. Chem..

[13] Seth A., Park H.S., Hong K.S. (2017). Current Perspective on In Vivo Molecular Imaging of Immune Cells. Molecules.

[14] de Almeida P.E., van Rappard J.R., Wu J.C. (2011). In vivo bioluminescence for tracking cell fate and function. Am. J. Physiol. Heart Circ. Physiol..

[15] Dothager R.S., Flentie K., Moss B., Pan M.H., Kesarwala A., Piwnica-Worms D. (2009). Advances in bioluminescence imaging of live animal models. Curr. Opin. Biotechnol..

[16] Badr C.E., Tannous B.A. (2011). Bioluminescence imaging: progress and applications. Trends Biotechnol..

[17] Serganova I., Moroz E., Cohen I., Moroz M., Mane M., Zurita J., Shenker L., Ponomarev V., Blasberg R. (2016). Enhancement of PSMA-Directed CAR Adoptive Immunotherapy by PD-1/PD-L1 Blockade. Mol. Ther. Oncolytics.

[18] Suchowski K., Pöschinger T., Rehemtulla A., Stürzl M., Scheuer W. (2017). Noninvasive Bioluminescence Imaging of AKT Kinase Activity in Subcutaneous and Orthotopic NSCLC Xenografts: Correlation of AKT Activity with Tumor Growth Kinetics. Neoplasia.

[19] Ponomarev V., Doubrovin M., Serganova I., Vider J., Shavrin A., Beresten T., Ivanova A., Ageyeva L., Tourkova V., Balatoni J. (2004). A novel triple-modality reporter gene for whole-body fluorescent, bioluminescent, and nuclear noninvasive imaging. Eur. J. Nucl. Med. Mol. Imaging.

[20] Bos P.D., Zhang X.H., Nadal C., Shu W., Gomis R.R., Nguyen D.X., Minn A.J., van de Vijver M.J., Gerald W.L., Foekens J.A., Massagué J. (2009). Genes that mediate breast cancer metastasis to the brain. Nature.

[21] Faustino-Rocha A., Oliveira P.A., Pinho-Oliveira J., Teixeira-Guedes C., Soares-Maia R., da Costa R.G., Colaço B., Pires M.J., Colaço J., Ferreira R., Ginja M. (2013). Estimation of rat mammary tumor volume using caliper and ultrasonography measurements. Lab Anim. (NY).

