# Supplementary material for: Introducing a new reporter gene, membrane-anchored Cypridina luciferase, for multiplex bioluminescence imaging
Source: Mol Ther Oncolytics. 2021 Mar 17;21:15–22. doi: 10.1016/j.omto.2021.03.004 (PMC8020342; doi:10.1016/j.omto.2021.03.004)
Supplement: Document 1. Figures S1 and S2 [file mmc1.pdf]

OMTO, Volume *21*

Supplemental information

Introducing a new reporter gene,

membrane-anchored *Cypridina* luciferase,

for multiplex bioluminescence imaging

Maxim A. Moroz, Juan Zurita, Anna Moroz, Ekaterina Nikolov, Yury Likar, Konstantin Dobrenkov, Jason Lee, Larissa Shenker, Ronald Blasberg, Inna Serganova, and Vladimir Ponomarev

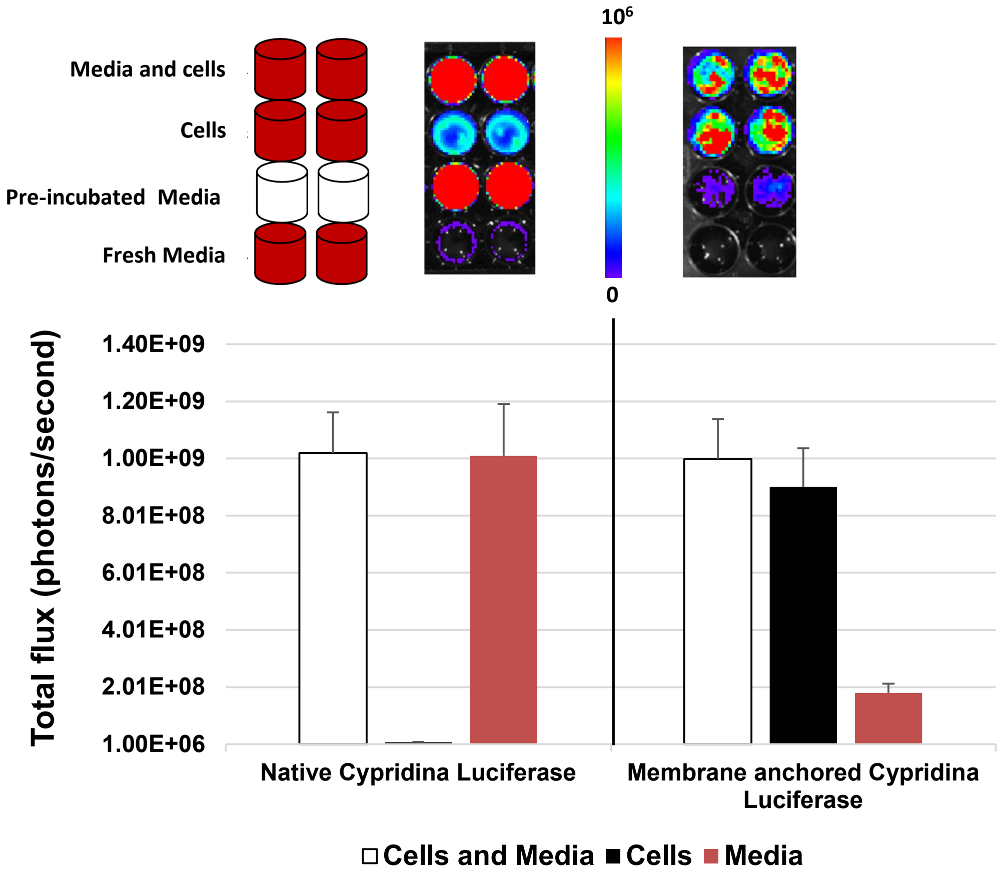

Supplemental figure 1. The effect of cell-surface anchoring of native Cypridina Luciferase on bioluminescence intensity. Bioluminescence signal output was measured on an IVIS-Spectrum analyzer. Cells + media (open bars); cells only (black bars) and post-incubation media only (red bars).

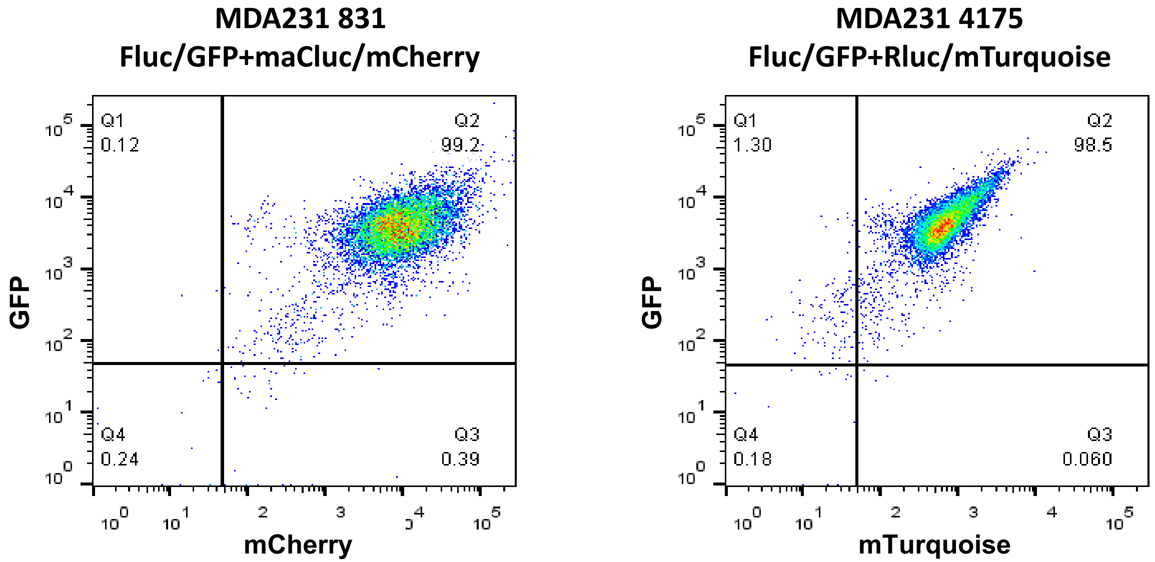

Supplemental figure 2. Post-sorting Flow Cytometry (FACS) profiles for MDA231/831 Fluc/GFP+maCluc/mCherry and MDA231/4175 Fluc/GFP+Rluc/mTurquoise cell lines demonstrating pure, double positive populations (A). Signal to noise (animals injected with substrate) ratios for in vivo BLI imaging of xenografts transduced with different luciferases. n=9 (B)
